# Antitumor and proliferative modulation by N- and O-heterocycles: a comprehensive review of cell growth regulation

**DOI:** 10.3389/fchem.2026.1861312

**Published:** 2026-09-03

**Authors:** Nurzhan Chinibayeva, Zulfiya Unerbaeva, Daniil Shepilov, Gulzat Berganayeva, Jichang Liu, Xin Pu, Akmaral Nurmahanova, Gulbanu Zhaksylykova, Sandugash Sydykbayeva, Moldyr Dyusebaeva

**Affiliations:** 1 Department of Chemistry, Faculty of Natural Sciences and Geography, Abai Kazakh National Pedagogical University, Almaty City, Kazakhstan; 2 Faculty of Chemistry and Chemical Technology, Farabi University, Almaty City, Kazakhstan; 3 Key Laboratory for Green Processing of Chemical Engineering of Xinjiang Bingtuan, School of Chemistry and Chemical Engineering, Shihezi University, Shihezi, China; 4 Faculty of Biology and Biotechnology, Farabi University, Almaty City, Kazakhstan; 5 Faculty of Natural and Technical Sciences, Zhetysu University Named After Ilyas Zhansugurov, Taldykorgan, Kazakhstan

**Keywords:** anticancer therapeutic, antitumor agents, heterocyclic chemistry, hydrazides, nitrogen-containing heterocycles, oxygen-containing heterocycles, sustainable healthcare

## Abstract

The persistence of resistance, recurrence, and limited selectivity in cancer therapy necessitates the development of structurally advanced antitumor agents capable of modulating multiple intracellular pathways. This review provides a systematic analysis of nitrogen- and oxygen-containing heterocycles as key platforms in anticancer drug design, with an emphasis on integrated molecular architectures combining multiple pharmacophoric fragments. A central and unifying principle emerging from the analyzed studies is the decisive role of hydrazide–hydrazone functionalization as a pharmacophoric enhancer, which should be considered not merely as a substituent but as a core structural element governing conformational flexibility, electronic distribution, and target-binding efficiency. Its incorporation consistently correlates with enhanced antiproliferative activity, apoptosis induction, and improved selectivity across diverse tumor models. Within this framework, the most efficient representatives highlight the practical significance of this design strategy: compound 17 demonstrates exceptional activity with GI_50_ values of 0.01–0.1 μM among N-heterocyclic systems, while compound 40 emerges as a leading O-heterocyclic analogue, exhibiting IC_50_ values of 0.45 μg/mL against MCF-7 cells. Collectively, these findings support the strategic prioritization of hydrazide-centered heterocyclic scaffolds as a foundation for the rational development of next-generation anticancer therapeutics.

## 1 Introduction

Despite the remarkable advances achieved in modern oncology, the perception of cancer as a uniformly controllable disease remains fundamentally flawed. While therapeutic success has been achieved for selected malignancies, a substantial proportion of tumor types continue to exhibit resistance, recurrence, and limited responsiveness to existing treatments. This persistent clinical challenge underscores the necessity for the continued development of structurally innovative compounds capable of modulating proliferative signaling and overcoming adaptive resistance mechanisms. Within this landscape, heterocyclic chemistry has retained a central and increasingly influential role in anticancer drug discovery. Nitrogen- and oxygen-containing heterocycles, in particular, stand out due to their exceptional structural versatility and reproducible biological relevance (Bashandy et al., 2011; Ghorab and Al-Said, 2012; Samir and Mohareb, 2022). Their capacity to support diverse functionalization patterns and engage in multiple intermolecular interactions renders them highly suitable for the rational design of bioactive molecules with enhanced pharmacological profiles.

Contemporary research further highlights the significance of integrating multiple heterocyclic motifs within unified molecular frameworks (Berillo and Dyusebaeva, 2022; Dyusebaeva et al., 2017; Dyusebaeva and Kalugin, 2015). Architectures incorporating hydrazide, chromene, dihydropyridine, and sulfone fragments exemplify how structural complexity can be strategically leveraged to achieve tunable and amplified biological responses. Such hybrid systems reflect an advanced design philosophy, where synergistic interactions between pharmacophoric elements contribute to improved efficacy and broadened functional potential (Yu et al., 2018; Yu et al., 2023; Yu et al., 2025). Equally important is the recognition that these compounds frequently exert their effects through coordinated, multi-level mechanisms. Beyond direct cytotoxicity, they demonstrate the capacity to influence intracellular homeostasis, including the modulation of oxidative and metabolic processes. This integrative mode of action aligns with current paradigms in anticancer therapy, which increasingly favor compounds capable of regulating complex biological networks.

Nitrogen- and oxygen-containing heterocycles represent a dynamic and continuously evolving domain that consistently reaffirms its significance in medicinal chemistry. Their recurring success across diverse studies positions them as a foundational platform for the development of next-generation antitumor agents. Accordingly, this review examines how quinoline, indole, chromene, furan, coumarin, and related heterocyclic cores regulate anticancer activity through their substitution patterns, linker geometry, and target associations. Particular attention is given to distinguishing target-determining heterocyclic features from auxiliary groups that primarily modulate molecular orientation, physicochemical properties, or cellular availability.

## 2 N-heterocycles

**FIGURE 1:**
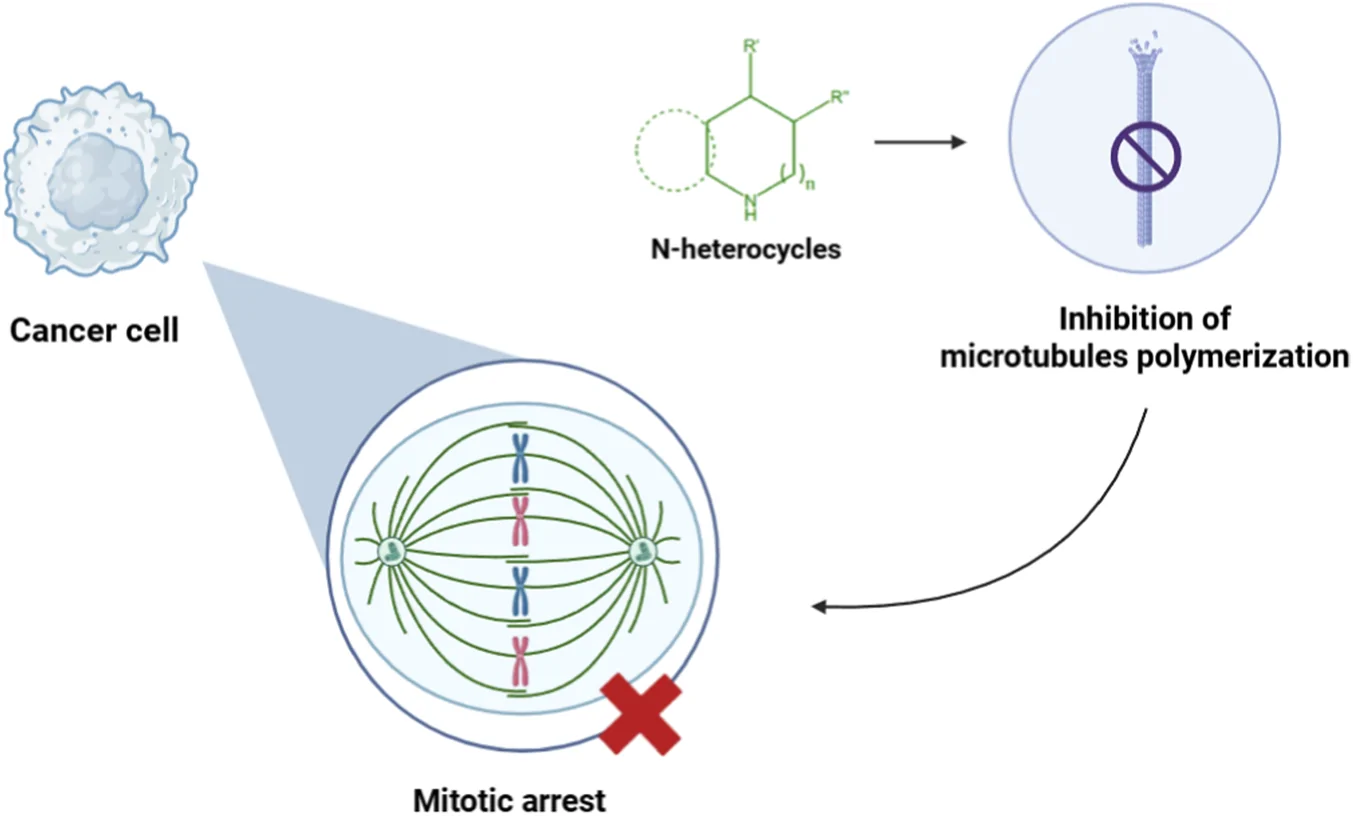
Mechanism of antitumor activity of N-heterocycles.

The anticancer activity of N-heterocyclic compounds is largely associated with their ability to interact with intracellular targets that regulate mitosis, DNA processing, and survival signaling (Yancheva et al., 2024). Owing to the presence of nitrogen atoms within the heterocyclic framework, these scaffolds readily establish donor–acceptor and hydrogen-bonding interactions with enzymes and structural proteins involved in tumor cell proliferation, which explains their frequent activity as kinase modulators, topoisomerase-directed agents, and tubulin-targeting compounds. In mechanistic terms, many N-heterocycles suppress cancer cell growth by interfering with microtubule dynamics, arresting the cell cycle, and subsequently triggering apoptosis (Kumar et al., 2023; Figure 1). This behavior becomes even more pronounced after incorporation of a hydrazide–hydrazone fragment, which increases pharmacophoric adaptability and expands the range of target interactions.

### 2.1 Quinoline and fused quinoline derivatives

Quinoline-based derivatives have consistently demonstrated pronounced antiproliferative potential across diverse tumor models, reinforcing the perception of this heterocyclic scaffold as a structurally privileged platform in anticancer drug design. In this context, the synthesis and biological evaluation of a series of quinoline derivatives revealed a distinctly heterogeneous cellular response, where HT29 human colon cancer cells exhibited relative resistance, while MDA-MB231 breast cancer cells remained susceptible across the entire compound panel, with IC_50_ values ranging from 4.6 to 48.2 μM (Arafa et al., 2013). Within this framework, Schiff’s base 1 emerged as a particularly compelling candidate, exhibiting IC_50_ values of 4.7 μM against HT29 and 4.6 μM against MDA-MB231, with its activity mechanistically linked to inhibition of proliferation and induction of cytotoxicity, as confirmed by luminescence-based assays. These observations not only underscore the intrinsic bioactivity of quinoline scaffolds but also highlight the importance of cellular context in modulating therapeutic response.

Expanding this paradigm, the antiproliferative capacity of quinoline-containing systems was further substantiated through the evaluation of a structurally diverse library of ninety tetrahydroquinoline derivatives against HepG2 and A549 cell lines (Fathy et al., 2020). Notably, compounds 2a, 2b, and 2c demonstrated remarkable potency toward HepG2 cells, with IC_50_ values of 1.1, 0.8, and 0.53 μM, approaching the efficacy of doxorubicin (IC_50_ = 0.4 μM), while compounds 3a and 3b displayed strong activity against A549 cells with IC_50_ values of 0.69 and 1.06 μM, respectively, relative to 5-fluorouracil (IC_50_ = 0.6 μM). The mechanistic profile of these derivatives revealed the induction of apoptosis and cell-cycle arrest, with structure–activity relationship analysis emphasizing the critical role of hydrazide and pyrazole pharmacophores in enhancing cytotoxic performance. The observed higher sensitivity of A549 cells further suggests that subtle variations in intracellular uptake or molecular target accessibility may significantly influence the biological outcome, thereby opening avenues for targeted optimization.

A more functionally refined strategy involving 2-(quinoline-4-carbonyl)hydrazide derivatives bearing acrylamide fragments further illustrates the capacity of quinoline systems to engage specific molecular targets (Abd El-Lateef et al., 2024). Compounds 4a, 4b, and 4c exhibited pronounced cytotoxicity against MCF-7 cells, with IC_50_ values of 3.39, 5.94, and 2.71 μM, respectively, surpassing doxorubicin (IC_50_ = 6.18 μM). Of particular significance, compound 4c demonstrated potent EGFR kinase inhibition (IC_50_ = 0.22 μM), closely matching lapatinib (IC_50_ = 0.18 μM), thereby positioning this derivative as a dual-acting agent. Mechanistic investigations further revealed that compounds 4a-c induced G1-phase arrest and promoted apoptosis through substantial upregulation of p53 and caspase-9 (7.4- and 8.7-fold increases, respectively), indicating a coordinated disruption of proliferative signaling and apoptotic control. Such findings reflect a transition from general cytotoxicity toward mechanism-driven molecular targeting within quinoline-based systems.

The versatility of quinoline hydrazide-hydrazone frameworks is further exemplified in neuroblastoma models, where compounds 5 and 6 exhibited potent and selective cytotoxicity (Bingul et al., 2016). Compound 6, in particular, demonstrated IC_50_ values of 2.9 μM and 1.3 μM against SH-SY5Y and Kelly cell lines, respectively, while compound 5 showed IC_50_ values of 5.7 μM and 2.4 μM. In contrast, only moderate activity was observed in breast cancer models, with IC_50_ values of 14.1 μM (MCF-7) and 18.8 μM (MDA-MB-231), underscoring a notable degree of tumor-type selectivity. Importantly, both compounds displayed preferential activity toward malignant neuroblastoma cells over normal human lung fibroblasts, suggesting a favorable therapeutic index. Mechanistically, compound 6 induced G1-phase arrest via upregulation of p27kip1, indicating a cytostatic mechanism that complements classical cytotoxic pathways and broadens the functional scope of these derivatives.

Further structural diversification through benzotriazole-substituted quinoline hydrazones provided additional insight into structure–activity relationships governing cytotoxic potency (Korcz et al., 2018). Compounds 7 and 8, incorporating a 4-chlorophenyl substituent, exhibited the most pronounced activity against DAN-G, LCLC-103H, and SISO cell lines, with IC_50_ values ranging from 2.29 ± 0.86 to 3.55 ± 2.44 μM. The enhancement of activity was attributed to the synergistic contribution of the benzotriazole scaffold and the electron-withdrawing 4-chlorophenyl fragment, while increased lipophilicity further improved cellular penetration and overall efficacy. These findings emphasize the importance of fine structural tuning in maximizing the pharmacological potential of quinoline-based hydrazones.

**FIGURE 2:**
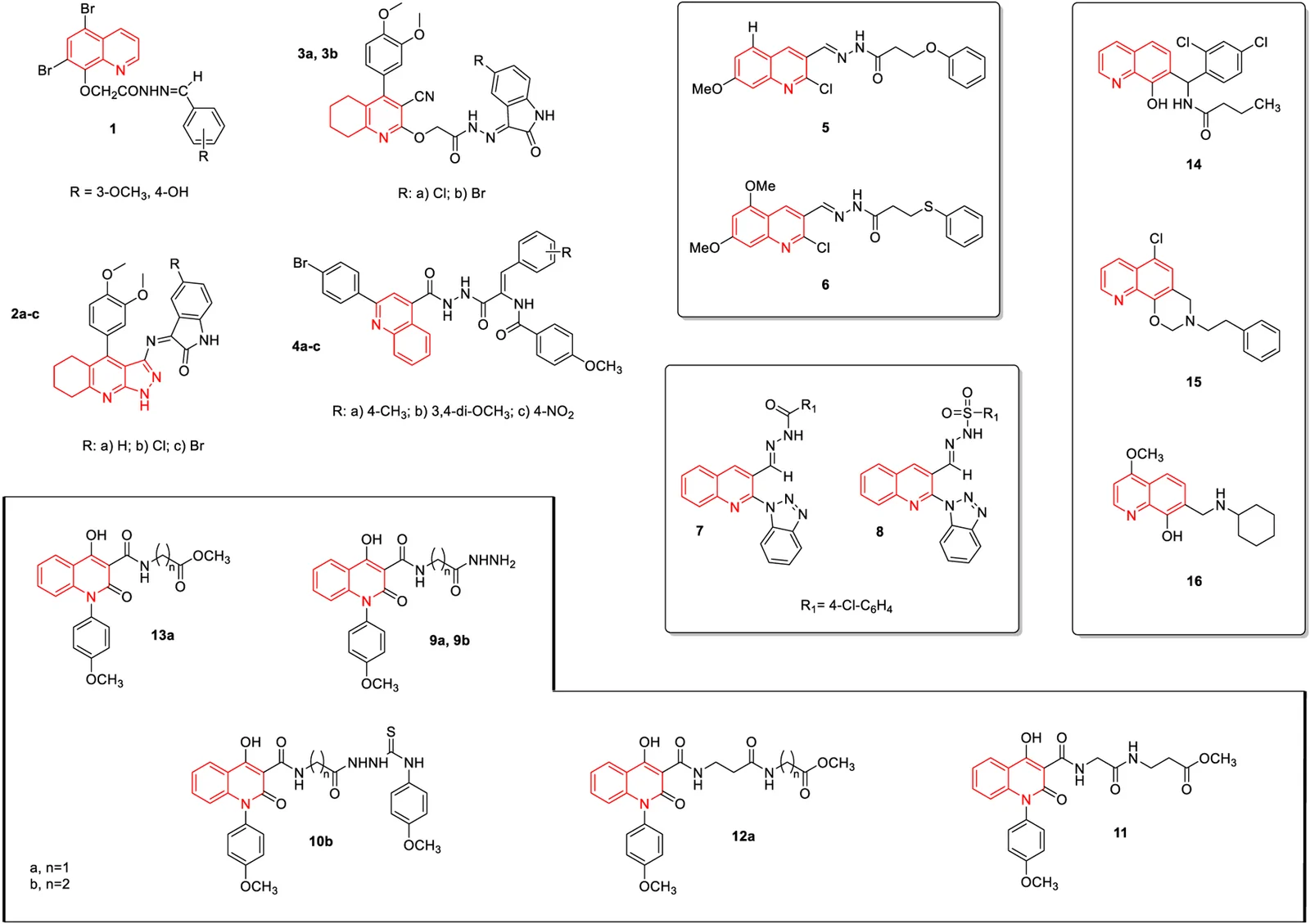
Novel quinoline- and fused quinoline-based compounds.

The exploration of 2-oxoquinoline hydrazide derivatives highlights an emerging trend toward multifunctional agents that integrate anticancer and antioxidant (Mohamed et al., 2023). Within this series, compound 9a exhibited notable antioxidant activity against DPPH radicals (IC_50_ = 18.8 μM), while compound 10b showed moderate activity (IC_50_ = 26.89 μM). In terms of anticancer efficacy, compound 9b demonstrated the highest activity against HCT-116 cells (IC_50_ = 18.8 μM), whereas structural variations in peptide fragments significantly influenced activity, as evidenced by compound 11 (IC_50_ = 27.28 μM) outperforming its analogue 12a (IC_50_ = 61.4 μM). Additionally, compound 13a displayed the most pronounced H_2_O_2_ scavenging capacity (IC_50_ = 38.7 μM). Collectively, these data suggest that quinoline-based hydrazides may provide a useful platform for the development of dual anticancer and antioxidant agents. A complementary target-directed strategy, independent of hydrazide–hydrazone functionalization, was demonstrated for 8-hydroxyquinoline and oxazino-quinoline derivatives identified as negative modulators of GLI transcription factors. The initial 8-hydroxyquinoline hit reduced GLI1 protein levels and inhibited the proliferation of DAOY medulloblastoma and A375 melanoma cells, with IC_50_ values of 0.19 and 0.11 μM, respectively. Focused structural modification showed that the biological response depended on both the substitution pattern and conformational organization of the quinoline scaffold: 3,4-dimethoxyphenyl substitution preserved submicromolar activity, whereas methylation at C3 or removal of essential hydrophobic groups diminished GLI1 modulation. Rigidification of the C7–C8 region into an oxazino-quinoline system also retained activity and provided a basis for subsequent optimization (Manetti et al., 2022). Development of these scaffolds subsequently produced compounds 14, 15, and the structurally simplified 4-methoxy-8-hydroxyquinoline – 16. These compounds inhibited GLI1/GLI2-dependent transcription and suppressed the growth of several GLI-activated cancer cell lines, with IC_50_ values of 0.1–1.6 μM. Mechanistic experiments demonstrated that compound 16 interfered with the recruitment of GLI1 and GLI2 to target DNA, while genetic depletion of either transcription factor reduced cellular sensitivity to the compounds, supporting target-dependent activity. Compound 16 additionally induced caspase-associated apoptosis and showed preferential activity toward malignant rather than non-neoplastic cells. In an A375 melanoma xenograft model, administration of compound 16 at 25 mg/kg twice daily reduced tumor volume by approximately 70% without evident systemic toxicity, although its short half-life and rapid clearance indicate the need for further pharmacokinetic optimization (Maresca et al., 2023). The studies demonstrate that the quinoline scaffold can support target-directed anticancer activity independently of hydrazide–hydrazone functionalization. All the mentioned compounds of this section presented on Figure 2.

### 2.2 Indole-, bis(indolyl)-, and related fused indole derivatives

Indole-based scaffolds consistently emerge as structurally privileged frameworks, reflecting their intrinsic ability to interfere with key regulatory processes governing tumor cell survival and proliferation. Their biological relevance is strongly associated with apoptosis induction, disruption of cell-cycle progression, and modulation of microtubule dynamics, positioning these systems at the intersection of multiple anticancer mechanisms. Within this context, indolylhydrazones and benzimidazole-hydrazone derivatives provide a compelling illustration of this activity, where compound 17 demonstrated pronounced submicromolar cytotoxicity across a broad spectrum of tumor cell lines, with GI_50_ values ranging from 0.01 to 0.1 μM and a mean GI_50_ of 0.039 μM (Acar Çevik et al., 2018). Notably, its marked selectivity toward the COLO 205 colon cancer cell line, supported by GI_50_, TGI, and LC_50_ values of 0.018, 0.034, and 0.063 μM, respectively, underscores the capacity of indole-based hydrazones to achieve both potency and selectivity within a single molecular framework.

This mechanistic versatility is further reinforced by studies on indole hydrazide derivatives, where compound 18 exhibited the highest antiproliferative activity against MCF-7 cells, with an IC_50_ value of 3.01 μM (Kilic-Kurt et al., 2020). Its biological action was associated with G0/G1-phase arrest and a coordinated modulation of apoptotic signaling pathways, including upregulation of Bax, downregulation of Bcl-2, and reduction of procaspase-3 and procaspase-9 levels, collectively indicating activation of a caspase-dependent apoptotic cascade. The additional observation of DNA damage, evidenced by increased H2AX and ATM phosphorylation, further highlights the multifaceted mechanism of action inherent to these systems, demonstrating that indole hydrazide derivatives can simultaneously engage multiple intracellular targets.

The structural and functional adaptability of indole-based systems becomes even more apparent in bis(indolyl)hydrazide–hydrazone derivatives, where compound 19a displayed significant cytotoxicity against MCF-7 and MDA-MB-231 cell lines, with IC_50_ values of 8.3 and 5.1 μM, respectively (Kumar et al., 2012). Mechanistic analysis revealed that this activity is closely linked to inhibition of tubulin polymerization, leading to G2/M-phase arrest and subsequent apoptosis. Importantly, structural modifications such as N-methylation, exemplified by compound 19d, resulted in enhanced cytotoxicity toward LnCaP cells, indicating that even subtle chemical variations can substantially influence biological performance. Within this framework, the hydrazide–hydrazone linkage represents one of several structural features that may contribute to activity, together with the indole core, substitution pattern and target-binding properties, reinforcing its role as a key pharmacophoric element.

A more advanced level of structural optimization is illustrated by compound 20, which represents a leading bis(indolyl)-hydrazide–hydrazone derivative with potent activity against A549 cells (IC_50_ ≈ 2 μM) while maintaining significantly lower toxicity toward normal WI38 fibroblasts and PBMC (IC_50_ values of 48.5 and 62 μM, respectively) (Das Mukherjee et al., 2016). Its mechanism involves strong tubulin binding, inhibition of tubulin assembly, G2/M-phase arrest, mitochondrial depolarization, and apoptosis induction following microtubule depolymerization. These features collectively highlight the ability of optimized indole-based hydrazones to combine high potency with favorable selectivity profiles. This trend is further supported by the work of Saruengkhanphasit et al. (2021), where structural refinement of compound 21a led to highly active derivatives such as 21b, 21c, and 21d, all exhibiting IC_50_ values below 0.5 μM against HuCCA-1 cells, with compound 21b reaching 0.34 μM against HepG2 and compound 21d showing 0.43 μM against A549. Notably, compound 22 demonstrated the highest activity against A549 (IC_50_ = 0.19 μM), despite weaker tubulin inhibition, suggesting that alternative or complementary mechanisms may contribute to its biological effect.

The functional diversity of indole hydrazones is further expanded by compounds 23, 24, and 25, which exhibited submicromolar antiproliferative activity against K562 and Colo-38 cell lines (Demurtas et al., 2019). Among these, compounds 24 and 25 stand out as multifunctional leads, combining strong cytotoxicity with antioxidant and photoprotective properties, thereby illustrating the potential of indole-based systems to integrate multiple therapeutic functions within a single molecular entity. Additional support for the high pharmacological potential of these scaffolds is provided by compound 26, which demonstrated superior activity against MCF-7 cells (IC_50_ = 2.73 ± 0.14 μM), surpassing staurosporine, and induced 39.26% apoptosis alongside pronounced S-phase arrest (Salama et al., 2023). Its ability to inhibit multiple kinases, including PI3K isoforms, CDK2, AKT-1, and EGFR, coupled with significant tumor volume reduction *in vivo*, emphasizes the emergence of multitarget-directed ligands within this class.

**FIGURE 3:**
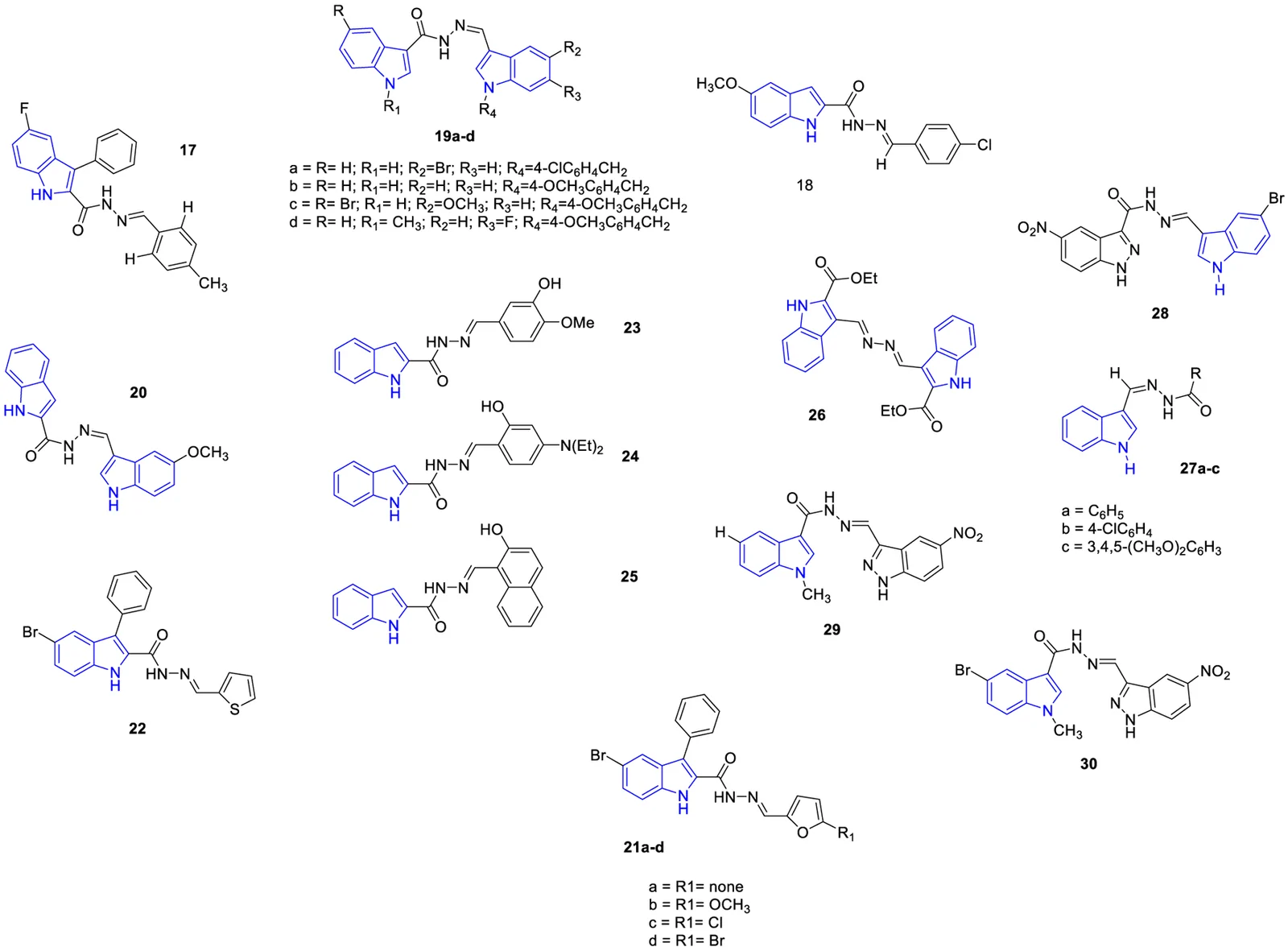
Indole-, bis(indolyl)-, and related indole derivatives.

The high potency of indolyl hydrazide–hydrazone derivatives is further confirmed by compounds 27b and 27c, which exhibited IC_50_ values of 0.42 μM and 0.8 μM, respectively, along with a remarkable 173-fold selectivity for MCF-7 cells in the case of compound 27b (Sundaree et al., 2016). Their mechanism, involving apoptosis induction through PARP1 cleavage, reinforces the central role of programmed cell death pathways in mediating their anticancer effects. The evaluation of compound 28 and related derivatives demonstrated strong cytotoxicity against MCF-7 cells (IC_50_ = 1.93 μM), with additional compounds 29 and 30 retaining significant activity (Sreenivasulu et al., 2017). The selective inhibition of cancer cells over normal HEK-293 cells, together with molecular docking evidence supporting tubulin polymerization inhibition, further consolidates the role of indole-based hydrazone systems as highly effective and selective modulators of tumor cell proliferation. All the mentioned compounds of this section presented on Figure 3.

### 2.3 Application of hydrazide–hydrazone derivatives as potentially more active antitumor compounds

Beyond the above-discussed compounds, where heterocyclic frameworks constituted the principal structural cores, the evolution of anticancer drug design increasingly reflects a transition from isolated structural motifs to integrated, functionally enriched systems. In this context, hydrazide–hydrazone derivatives emerge not merely as structural modifications, but as strategically significant elements that unify molecular flexibility, electronic adaptability, and biological responsiveness. Their widespread incorporation into heterocyclic scaffolds underscores a broader conceptual shift: the deliberate construction of multifunctional molecules capable of engaging diverse intracellular targets while maintaining selectivity toward malignant cells. This perspective not only highlights the synthetic attractiveness of hydrazone chemistry but also frames it as a central axis in the rational development of next-generation antitumor agents.

This structural paradigm is convincingly illustrated by hydrazide-hydrazone derivatives 31a-u (Akdağ et al., 2022), where compound 31h, bearing a pyrrole fragment, demonstrated outstanding cytotoxic activity with IC_50_ values of 1.3, 3.0, and 1.7 μM against PC-3, MCF-7, and HT-29 cell lines, respectively. Importantly, these effects were accompanied by exceptional selectivity indices of 181, 78.4, and 138.4, indicating a highly favorable therapeutic profile. Mechanistic analysis revealed apoptosis induction via caspase-3 activation and an increased proportion of early apoptotic cells, while pharmacokinetic and bioavailability assessments further emphasized the translational relevance of this compound. Thus, the integration of hydrazone functionality into heterocyclic systems can result not only in enhanced potency but also in a refined balance between efficacy and selectivity. Extending this observation, 4-benzyl-morpholine-2-carboxylic acid N'-[2-(4-benzoyl-phenoxy)-acetyl]-hydrazide derivatives (Al-Ghorbani et al., 2017) demonstrate how such structural features translate into robust biological performance across both *in vitro* and *in vivo* models. Within this series, compounds 32a and 32b exhibited the highest cytotoxicity, with compound 32a showing IC_50_ values of approximately 7.5 μM against DLA and 10.1 μM against A549 cells, and compound 32b displaying IC_50_ values of 9.3 μM against MCF-7 and 13.5 μM against A549 cells. Notably, their ability to reduce tumor volume and ascites secretion in murine models establishes a clear connection between molecular design and physiological efficacy. Their mechanism, involving caspase-activated DNase-mediated DNA fragmentation and G2/M phase arrest, further reinforces the concept that hydrazone-containing systems can effectively disrupt both proliferative signaling and genomic integrity. While high potency remains a central objective, the importance of controlled and targeted activity becomes evident in naproxen-derived 1,2,4-triazole hydrazide-hydrazone derivatives (Han et al., 2019). In this case, compound 33a exhibited moderate cytotoxicity, with IC_50_ values of 26.0 μM for PC3, 34.5 μM for DU-145, and 48.8 μM for LNCaP cells, yet demonstrated enhanced accumulation in prostate tissue. This observation highlights an essential dimension of modern anticancer research: the capacity to engineer molecules that balance activity with tissue specificity. Compounds 33b, 33c, and 33d, with IC_50_ values ranging from 43.0 to 49.8 μM, further illustrate how subtle structural variations influence biological response, emphasizing the tunability of hydrazone-based systems.

This principle of structural tuning is further reinforced by pyridine-2,6-dicarbohydrazide derivatives (Şenkardeş et al., 2021), where compounds 34a, 34b, and 34c exhibited selective cytotoxicity toward HT-29 and ISH cell lines. Compound 34a demonstrated IC_50_ values of 6.78 μM for HT-29 and 10.23 μM for ISH cells, while compound 34b achieved an IC_50_ of 8.26 μM against ISH cells. Importantly, these compounds maintained significantly higher IC_50_ values in NIH 3T3 fibroblasts, indicating reduced toxicity toward normal cells. Their mechanism, involving caspase-3 activation and apoptosis-associated morphological changes, further supports the idea that hydrazone-containing systems are capable of selectively engaging programmed cell death pathways.

Moving toward broader structural diversity, carbohydrazide-containing systems (Refat and Fadda, 2015) illustrate how the combination of multiple pharmacophoric fragments can amplify biological activity. In this study, compound 35b exhibited superior cytotoxicity compared to 5-fluorouracil, with IC_50_ values of 2.16, 4.86, and 5.90 μg/mL against HepG2, HCT-116, and MCF-7 cells, respectively. Additional compounds, including 35a, 35c, 35d, and 35e, also demonstrated strong activity. Structure–activity relationship analysis revealed that the presence of N-phenyl-substituted pyrazole rings, hydroxyl-containing pyran moieties, and isoxazole fragments significantly enhances cytotoxic performance, emphasizing the cumulative effect of multiple functional groups within a single molecular architecture.

At the same time, the balance between potency and safety remains a defining challenge, as illustrated by pyrrole hydrazones (Vladimirova et al., 2024). Compounds 36a and 36b exhibited selectivity indices of 2.11 and 3.83, respectively, surpassing cisplatin (SI = 0.38), while compound 36b displayed an IC_50_ of 44.63 ± 3.51 μM against SH-4 melanoma cells. Although their cytotoxicity is comparatively moderate, these results highlight an alternative design strategy focused on minimizing systemic toxicity while preserving biologically relevant activity. The progression toward structurally complex systems is further exemplified by fused heterocycles such as pyridazino[4,5-b]indole derivatives (Sarhan et al., 2020). Within this class, compound 37 exhibited an IC_50_ value of 4.25 μM against MCF-7 cells, exceeding that of 5-fluorouracil. Its mechanism, involving modulation of key apoptotic regulators and inhibition of the PI3K/AKT/mTOR pathway, supported by a binding energy of −18.87 kcal/mol toward PI3K, reflects a targeted and mechanistically refined mode of action. The observed *in vivo* efficacy further strengthens the translational potential of such systems. A comparable level of activity was observed for pyrroloquinoxaline hydrazide derivatives (Grande et al., 2009), where compounds 38a and 38b demonstrated submicromolar IC_50_ values and induced G0/G1 phase arrest. The interplay between reactive oxygen species generation and cell-cycle disruption highlights the multifactorial mechanisms that can be achieved through hydrazone-based design. In benzimidazole-hydrazone systems, compound 39b exhibited superior cytotoxicity against MCF-7 cells compared to cisplatin, while compound 39a demonstrated selective activity against lung carcinoma cells, further reinforcing the importance of scaffold-dependent selectivity.

**FIGURE 4:**
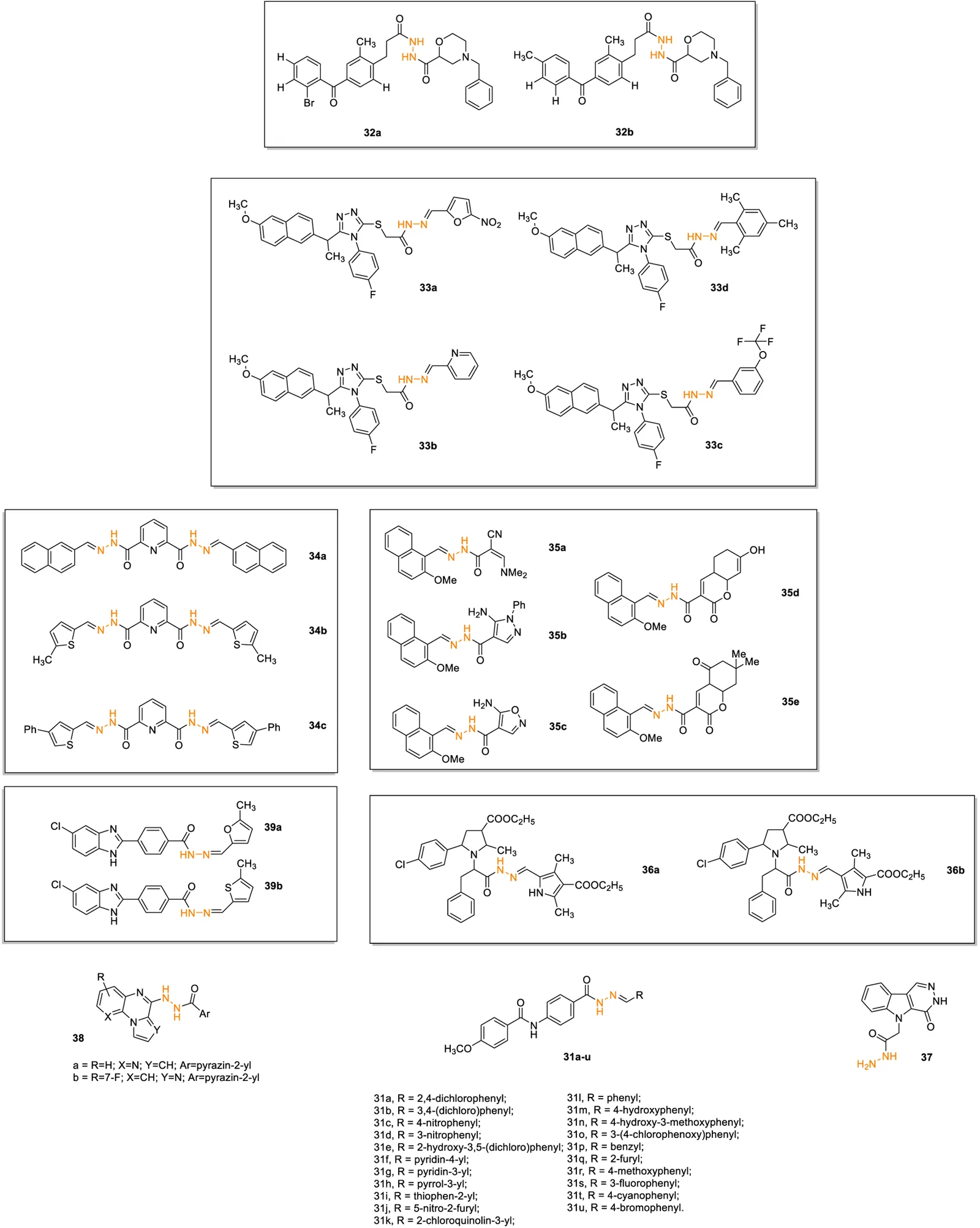
Hydrazide–hydrazone presence in novel derivatives.

This consistent trend is further supported by N'-(arylidene)-2-(arylamino)-6-(trifluoromethyl)nicotinohydrazide derivatives (Onnis et al., 2009), which demonstrated nanomolar activity across multiple tumor cell lines in the absence of observable *in vivo* toxicity, while NCI COMPARE analysis revealed a mechanism distinct from conventional cytotoxic agents, underscoring the emergence of alternative therapeutic pathways and reinforcing the potential of this scaffold for targeted applications. This divergence in mechanism aligns with observations in multifunctional hydrazone systems (Hagbani et al., 2023; Mahmoud et al., 2018), where coordinated disruption of cell-cycle progression, apoptosis induction, and DNA fragmentation define a multifaceted biological profile, with structure–activity relationship analysis highlighting the contribution of NH and SH groups in enhancing intermolecular interactions through hydrogen bonding and facilitation of DNA damage. A comparable level of functional adaptability is evident in hydrazone derivatives incorporating pyridine and isatin fragments (Zebbiche et al., 2021), where several compounds surpassed docetaxel in ovarian cancer models while maintaining strong antiproliferative activity in breast cancer cell lines, a trend that is further reinforced by morpholine-based derivatives (Taha et al., 2017) and azole-functionalized pyrazolo[3,4-b]pyridine systems (Nagender et al., 2016), collectively emphasizing the critical role of electronic modulation and targeted functional group optimization in refining cytotoxic performance and expanding the therapeutic applicability of hydrazone-containing heterocycles. All the mentioned compounds of this section presented on Figure 4.

## 3 O-heterocycles

**FIGURE 5:**
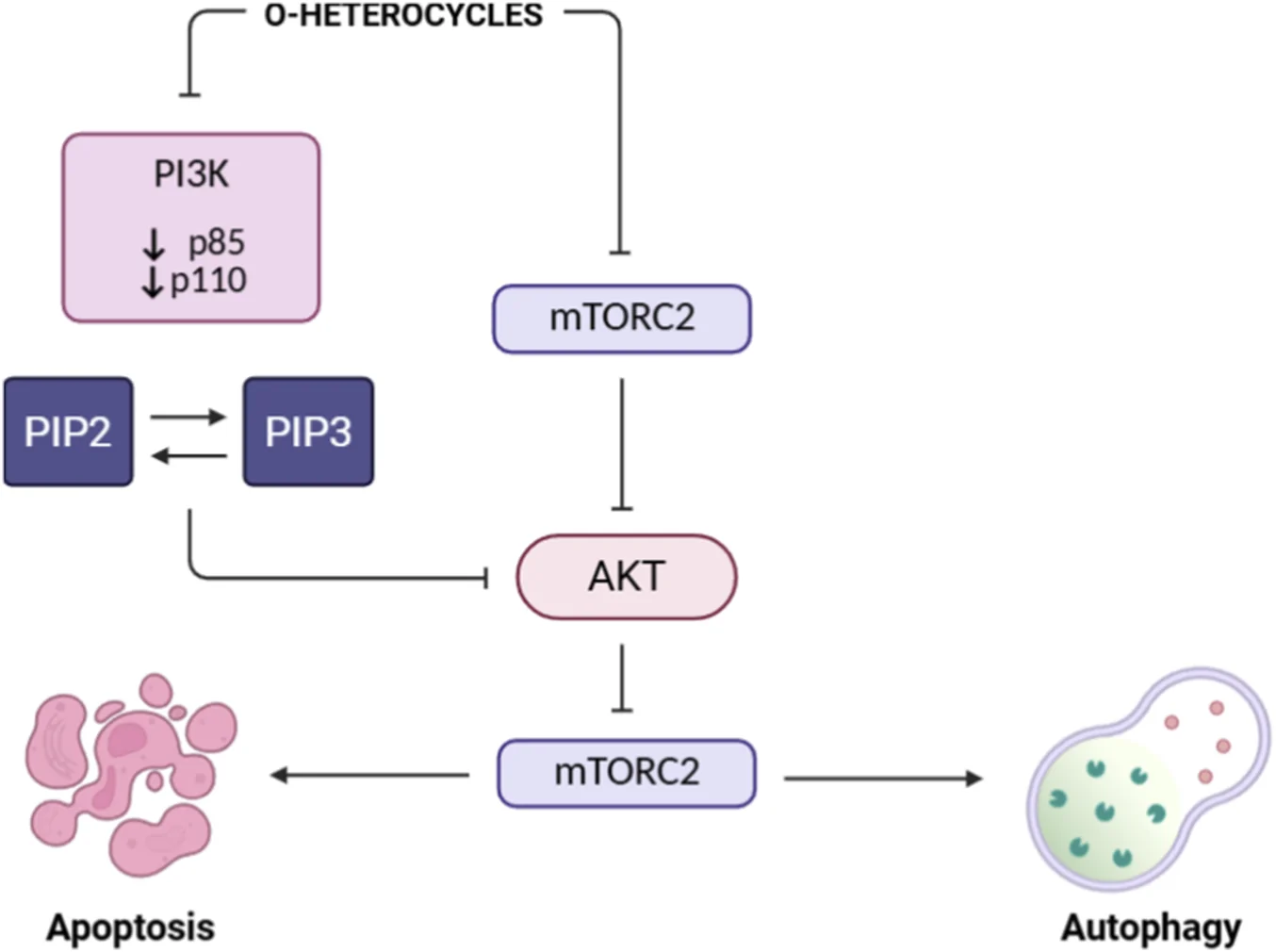
Mechanism of antitumor activity of O-heterocycles.

O-heterocyclic systems, particularly coumarins and related oxygen-containing fused scaffolds, exert anticancer effects through a broad spectrum of interconnected mechanisms involving apoptosis induction, suppression of proliferation, interference with migration and angiogenesis, and modulation of tumor-associated signaling pathways (Nasr et al., 2014; Shahbaz et al., 2024). Their oxygen-containing framework contributes to favorable electronic distribution and recognition properties that facilitate interaction with apoptosis-related and mitosis-related targets. In mechanistic studies, coumarin derivatives have repeatedly been associated with upregulation of pro-apoptotic signaling, including modulation of Bcl-2/Bax balance, cytochrome c release, caspase activation, and p53-associated cell-cycle disturbance; other reports also describe inhibition of proliferation and migration together with phase-specific cell-cycle arrest. Hydrazide–hydrazone linkers occur in selected derivatives, but their contribution depends on the O-heterocyclic framework to which they are attached. In particular, brominated coumarin hydrazide–hydrazone derivatives were reported to activate caspases 3/7 and induce apoptosis in drug-resistant Panc-1 pancreatic cancer cells, while related coumarin-based systems have also been linked to Cdc20 suppression and marked antiproliferative effects (Banikazemi et al., 2021). The mechanism of antitumor activity of O-heterocycles is presented on Figure 5.

Within the chromene-centered O-heterocyclic family, the strongest antiproliferative effects were concentrated in fused benzo[h]chromenes, chromeno[2,3-d]pyrimidines, and pyrano[3,2-c]chromenes. In the study by Alblewi et al., compound 40 (Alblewi et al., 2019), an aminoimino chromenopyrimidine derivative, emerged as the most potent analogue of the entire series, with IC_50_ values of 0.45 μg/mL against MCF-7, 0.7 μg/mL against HCT-116, and 1.7 μg/mL against HepG-2, while compound 41 and compound 42 also retained strong activity, particularly toward HCT-116 and HepG-2. These structural modifications were not only associated with high cytotoxicity but also with clear proliferation modulation, since compounds 41 and 42 caused marked accumulation of cells in the S and G2/M phases, whereas compound 40 produced the strongest G2/M arrest with concomitant reduction of the G1 population. Caspase-3/7 activity further confirmed apoptosis induction, reaching 13.46% and 17.36% in HCT-116% and 18.16% and 14.13% in HepG-2 for compounds 41 and 42, respectively, while DNA fragmentation, invasion suppression, and migration inhibition established that the most active fused chromenes extended their antitumor action beyond simple viability loss.

In a related pyrano[3,2-c]chromene study by El-Agrody et al. (2020), compounds 43a, 43b, and 43c displayed excellent activity against MCF-7, HCT-116, and HepG-2 with IC_50_ values spanning 0.2–1.7 μM, and these same derivatives induced G2/M-phase arrest together with apoptosis, confirming that heavily substituted pyranochromenes are likewise effective proliferation blockers. Additional work from El-Agrody et al. (2014) on benzo[h]chromene/chromeno[2,3-d]pyrimidine analogues identified compounds 40, 44, 45, and 46 as the most active members against MCF-7, HCT, and HepG-2, with compound 40 being the strongest against HepG-2, again reinforcing that lipophilic substitution and fused pyrimidine incorporation are decisive structural features for chromene-based antitumor optimization.

**FIGURE 6:**
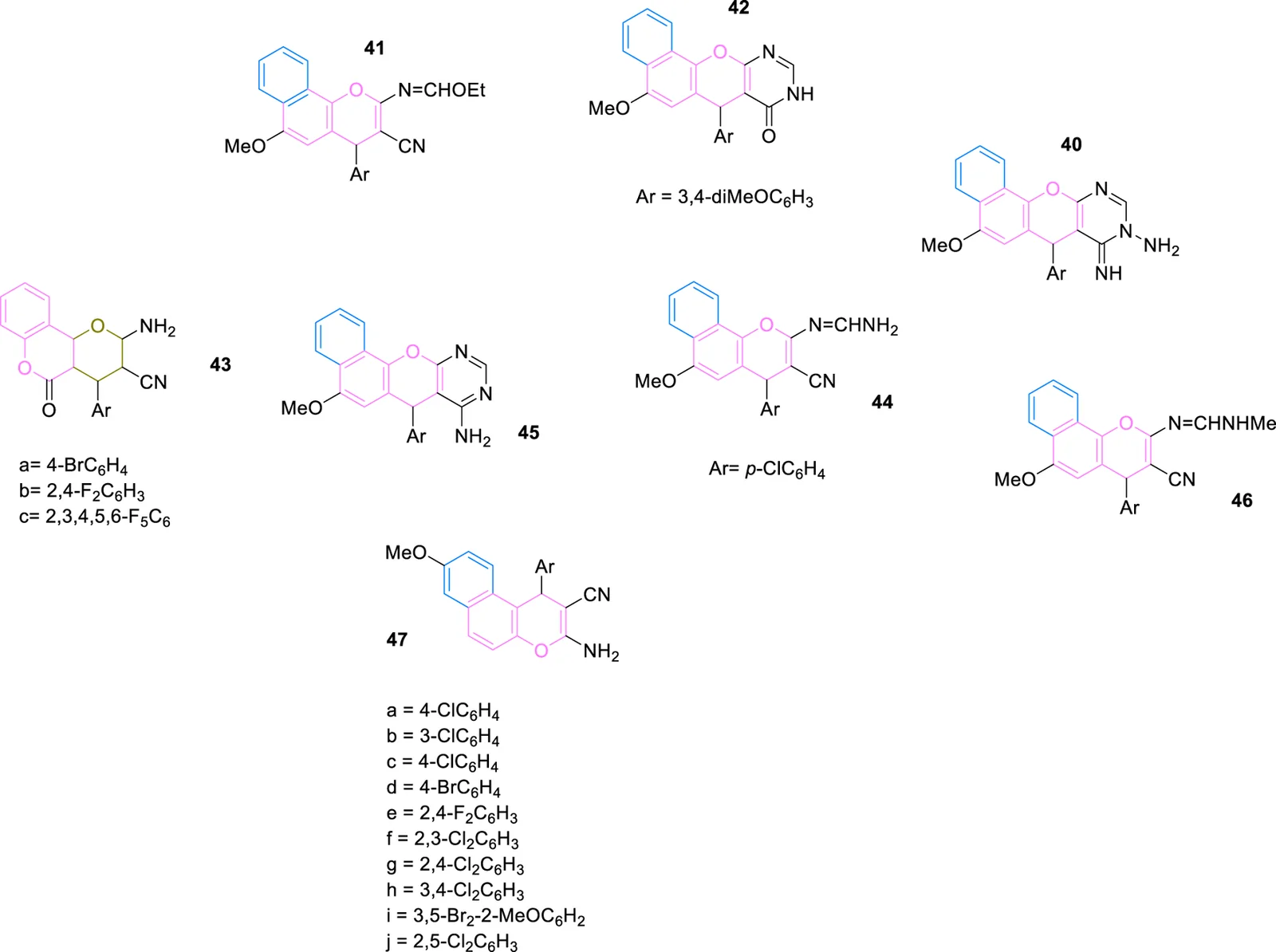
Chromene-centered O-heterocycles.

A second structurally distinct subgroup was formed by fused pyrans and benzo[f]chromene-derived pyran systems, in which the most active compounds combined strong cytotoxicity with robust apoptosis-associated signaling. In the recent benzo[f]chromene-2-carbonitrile pyran series (El-Wahab et al., 2024), compounds 47h, 47f, 47j, 47b, 47d, and 47i were highlighted as the most active against MCF-7, HCT-116, and HepG-2, whereas compounds 47a–i exhibited negligible toxicity toward HFL-1 and WI-38 normal cells. The recurring SAR trend across this pyran class was the positive impact of mono-, di-, and tri-halogenated phenyl substituents, which strongly suggests that halogen-driven lipophilicity and electronic withdrawal are key contributors to the cytotoxic enhancement of benzo[f]chromene-based pyrans. All the mentioned compounds of this section presented on Figure 6.

**FIGURE 7:**
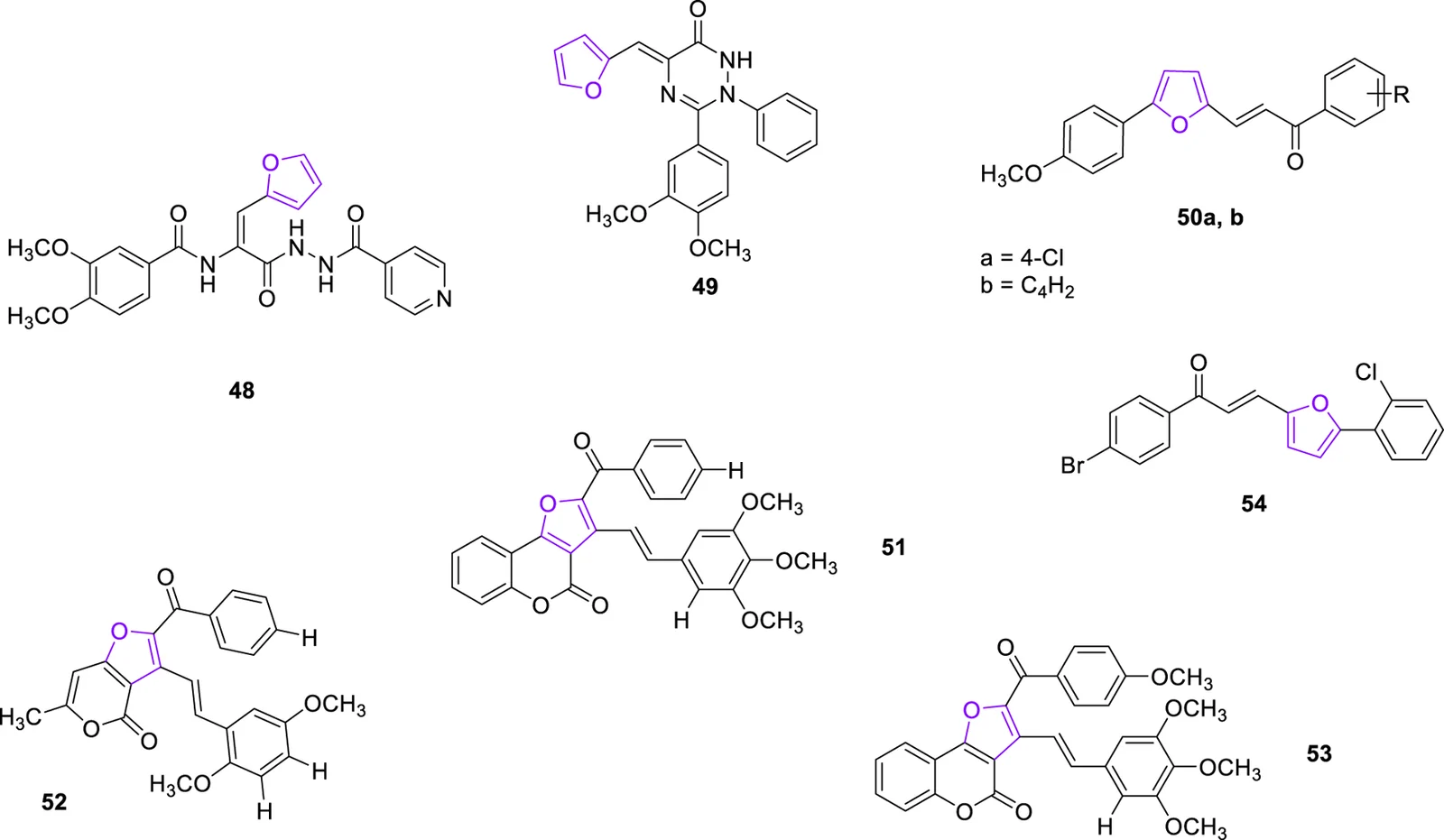
Novel furan-containing O-heterocycles.

Furan-containing O-heterocycles represented another highly productive structural class, but their biological output depended strongly on whether the furan ring was embedded in a direct chalcone system, a fused furo-pyran/chromenone framework, or a more complex spirooxindole hybrid. In the furan-based antimitotic series reported by Bukhari et al. (2022), compound 48 and compound 49 were the key cytotoxic leads against MCF-7, with IC_50_ values of 4.06 and 2.96 μM, respectively. Mechanistically, both compounds 48 and 49 produced G2/M arrest, pre-G1 accumulation, positive annexin V/PI staining, tubulin polymerization inhibition, and intrinsic mitochondrial apoptosis associated with increased p53 and Bax and reduced Bcl-2, indicating that in this scaffold the furan-containing motif acts as a genuine proliferation-modulating pharmacophore. In the breast cancer chalcone series of Solomon and Lee (2012), compound 50a was the most desirable furan-derived analogue because its antiproliferative effect was 3- to 7-fold greater in cancer cells than in the corresponding non-cancer breast line, while compounds 50b and 50a were the most active inhibitors of MDA-MB-231 and MDA-MB-468 proliferation. In the fused furo[3,2-c]pyran-4-one/furo[3,2-c]chromen-4-one family described by Rani (Rani et al., 2022), compound 51 was the most active overall, showing IC_50_ = 2.8 μM against MCF-7 and 3.8 μM against PC-3, while compounds 52 and 53 also retained good activity against MCF-7 with IC_50_ values of 6.9 and 5.3 μM. A further refinement of the furan-based design was presented by Altowyan et al. (2022), where the aryl-furan chalcone compound 54 was the most active compound in the whole set, with IC_50_ values of 4.1 ± 0.10 μM/mL against MCF7 and 3.5 ± 0.07 μM/mL against HepG2, thus outperforming staurosporine by 4.3-fold and 2.92-fold, respectively. In the same paper, docking suggested that the lead chalcone 54 may act through dual EGFR/CDK-2 inhibition, thereby linking the strongest furan-chalcone activity to a defined proliferation-associated signaling hypothesis. All the mentioned compounds of this section presented on Figure 7.

**FIGURE 8:**
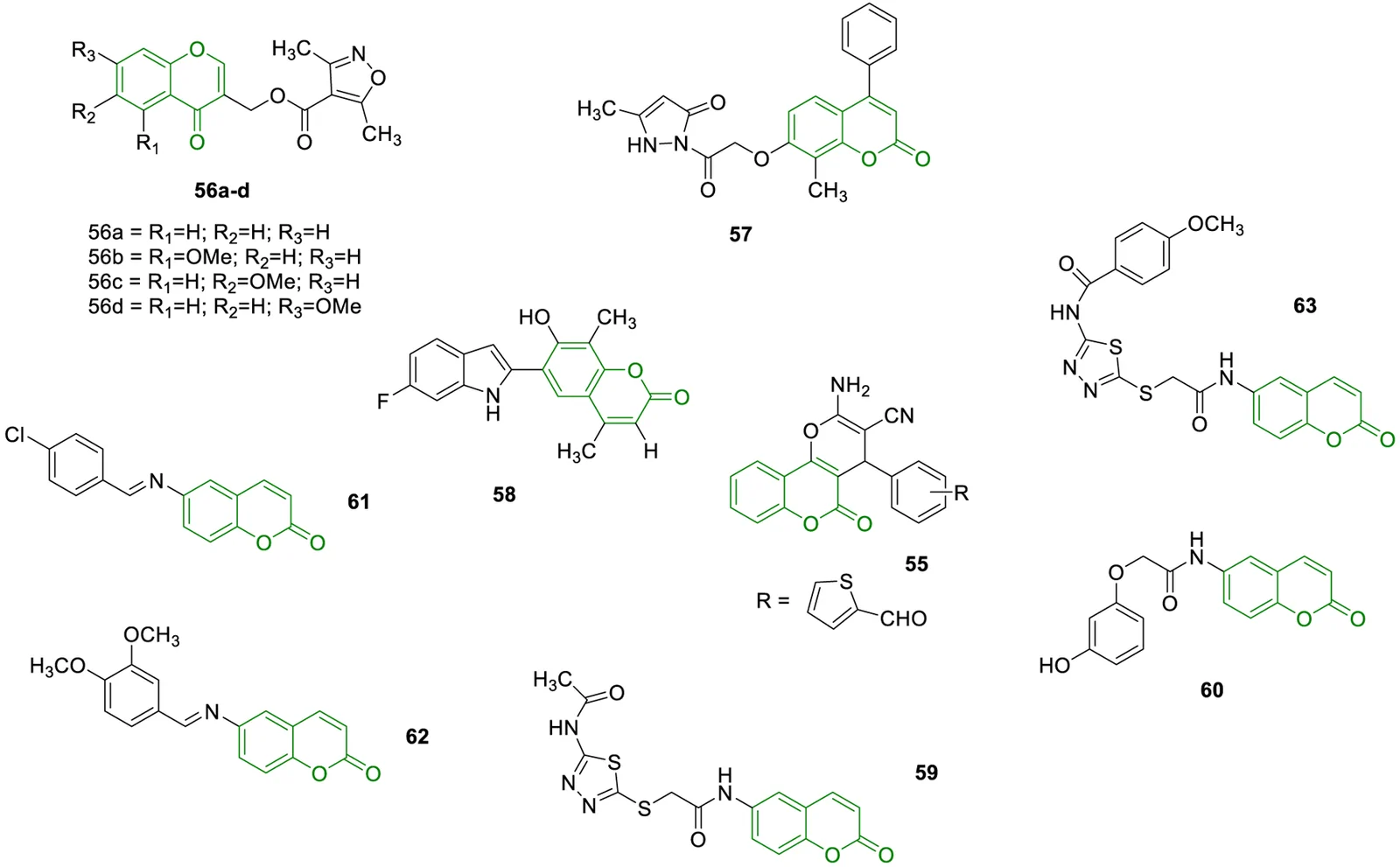
Novel coumarin-, benzopyran-4-one-, and coumarin-hybrid O-heterocycles.

Coumarin-, benzopyran-4-one-, and coumarin-hybrid O-heterocycles formed a broader but somewhat more heterogeneous structural block, in which the best compounds typically combined moderate-to-strong cytotoxicity with selectivity rather than extreme potency. In the study by Kumar et al. (2018), compound 55 was the lead 4-aryl/heteroaryl-4H-fused pyran, showing IC_50_ = 0.67 μM against HCT-116 and increasing the apoptotic population from 4.94% in the control to 39.68% at 1.5 μM, while also causing apoptotic body formation, mitochondrial membrane potential collapse, ROS generation, and cell-cycle arrest. This same compound 55 also inhibited tumor growth in Ehrlich ascites carcinoma, solid Ehrlich tumor, and sarcoma-180 models and remained non-toxic up to 1,000 mg/kg, making it one of the most mechanistically complete O-heterocyclic antitumor leads in the uploaded collection. In the benzopyran-4-one–isoxazole hybrid study of Gupta et al. (2023) compounds 56a–d were the clear leads, showing IC_50_ values of 5.2–22.2 μM against MDA-MB-231, whereas their IC_50_ values against HEK-293 normal cells were substantially higher, ranging from 102.4 to 293.2 μM, and compound 56a induced 50.8% apoptosis in MDA-MB-231 at 5 μM, thereby demonstrating that apoptosis induction contributes directly to the antiproliferative effect of this chromone–isoxazole construct. In the NCI-screened benzopyran-2-one hybrids reported by Ismail et al. (2010), compound 57, a pyrazolin-5-one-containing benzopyran-2-one, was the only analogue advanced to five-dose evaluation and showed a full-panel mean GI_50_ of 5.46 µM, TGI of 29.16 µM, and LC_50_ of 60.87 µM, with the best subpanel GI_50_ values in prostate cancer at 2.38 µM and CNS cancer at 2.58 µM, indicating broad-spectrum rather than highly line-selective growth suppression. In the 6-heteroarylcoumarin series (Galayev et al., 2015), compound 58, namely 6-(6-fluoro-1H-indol-2-yl)-7-hydroxy-4,8-dimethyl-2H-chromen-2-one, was the most active analogue, with mean GI_50_/TGI values of 3.28/13.24 µM and particularly strong sensitivity toward HOP-92, where GI_50_, TGI, and LC_50_ were 0.95, 4.17, and 29.9 µM, respectively, showing that incorporation of a 6-fluoroindole moiety into the coumarin core is a highly favorable structural modification. In the coumarin-thiadiazole study by Amin et al. (2018), the strongest contribution came not from exceptional potency but from mechanistic evidence of DNA interaction, since compounds 59 and 60 displayed the highest DNA/ethidium bromide quenching with KSV = 4.27 × 10^5^ M^-1^, and compounds 59, 61, and 62 showed dual DNA-binding behavior with methyl green displacement of 42%, 45%, and 43%, respectively, while compound 63 produced the best direct IC_50_ against HepG-2 at 24.9 μg/mL. All the mentioned compounds of this section presented on Figure 8.

## 4 Structure-activity relationship and future directions

Across the reviewed structures, anticancer activity can be interpreted through three cooperating molecular modules: a heterocyclic recognition core, a linker that controls spatial orientation, and peripheral groups that regulate electronic character and cellular exposure. The quinoline, indole, chromene, coumarin, or furan ring does not function independently, its biological direction is determined by how these modules are assembled. Table 1 summarizes the resulting relationships between structural features, molecular targets, and antiproliferative responses.

**TABLE 1:** Structural features and molecular targets of representative N- and O-heterocyclic compounds.

Compound/scaffold	Key structural determinant	Reported or proposed molecular target	Supporting evidence	Antiproliferative consequence	Main limitation	References
16	4-Methoxy-8-hydroxyquinoline with a preserved N/O recognition region	GLI1/GLI2	Transcriptional inhibition, impaired GLI–DNA recruitment, and reduced sensitivity after GLI depletion	Growth inhibition and caspase-associated apoptosis in GLI-dependent models	Short half-life and rapid clearance	Manetti et al. (2022); Maresca et al. (2023)
4c	Quinoline–acrylamide hybrid bearing a *para*-nitro substituent	EGFR	EGFR kinase inhibition (IC_50_ = 0.22 µM)	MCF-7 growth inhibition; apoptosis reported within the series	Kinase selectivity and cellular target engagement require validation	Abd El-Lateef et al. (2024)
19/20 series	Bis(indolyl) framework oriented by an acylhydrazone linker	Tubulin	Tubulin polymerization and binding assays	G2/M arrest, mitochondrial disruption, and apoptosis	Pharmacokinetic and *in vivo* validation remain limited	Das Mukherjee et al. (2016); Kumar et al. (2012)
37	Fused pyridazinoindole architecture	PI3K	Docking supported by PI3K/AKT/mTOR pathway modulation	MCF-7 growth inhibition, apoptosis, and *in vivo* activity	Direct target engagement has not been demonstrated	Sarhan et al. (2020)
48/49	Furan-containing conjugated antimitotic framework	Tubulin	Tubulin polymerization inhibition	G2/M arrest and mitochondrial apoptosis in MCF-7 cells	Binding site, broader selectivity, and pharmacokinetics remain unresolved	Bukhari et al. (2022)
54	Planar aryl–furan chalcone	EGFR/CDK2	Molecular docking only	Growth inhibition in MCF-7 and HepG2 cells	Biochemical target validation is absent	Altowyan et al. (2022)
55	Rigid 4-aryl/heteroaryl fused pyran	Molecular target	Cellular assays and efficacy in three *in vivo* tumor models	ROS generation, mitochondrial collapse, cell-cycle arrest, and apoptosis	The initiating molecular target remains unidentified	Kumar et al. (2018)
59–63	Planar coumarin–thiadiazole hybrid with heteroatom-rich substituents	DNA association	Ethidium-bromide quenching and methyl-green displacement	HepG2 growth inhibition; DNA affinity did not consistently correlate with cytotoxicity	Cellular relevance and selectivity of DNA binding remain unresolved	Amin et al. (2018)

The clearest recognition pattern is found among GLI-directed quinolines. The neighboring quinoline nitrogen and C8 oxygen form a compact donor–acceptor region capable of interacting with residues surrounding the GLI zinc-finger domain, while the C7 hydrophobic appendage stabilizes occupation of the adjacent nonpolar region. Disturbing this geometry through C3 methylation eliminated GLI1 modulation, whereas preserving the N/O arrangement during oxazino-ring formation retained activity. The peripheral aromatic group was favorable but not indispensable: sufficiently large cycloalkyl substituents could replace it, showing that hydrophobic volume, rather than aromaticity itself, governed this region of recognition. By contrast, the same substituent logic did not apply to EGFR-active quinolines. Here, a para-nitro group was more favorable than methyl or 3,4-dimethoxy substitution, whereas dimethoxy groups were well tolerated in the GLI series. Thus, electron withdrawal and methoxylation cannot be classified as generally beneficial or detrimental; their value depends on how they complement the electrostatic requirements of a specific target pocket (Abd El-Lateef et al., 2024; Manetti et al., 2022; Maresca et al., 2023).

The linker defines a second level of target orientation. In bis(indolyl) systems, the acylhydrazone group connects two aromatic domains while contributing carbonyl oxygen, imine nitrogen, and NH donor sites. This linear donor–acceptor arrangement repeatedly coincided with tubulin polymerization inhibition, suggesting that the hydrazone acts primarily as a positioning unit that presents the indole rings in a target-compatible geometry. It should not, however, be treated as a target-determining pharmacophore by itself. Similar hydrazone groups occur in compounds associated with PI3K signaling, DNA damage, or phenotypic apoptosis, demonstrating that the surrounding heterocycles ultimately define the biological direction. Compound 22 further exposes this limitation: strong cellular activity persisted despite weaker tubulin inhibition, indicating that optimization of the hydrazone-containing architecture changed cellular exposure or introduced an additional mechanism rather than simply strengthening tubulin binding.

Small heteroatom changes can redirect this preference. Replacement of the furan unit in 39a by the more polarizable thiophene ring in 39b enhanced activity toward MCF-7 cells, whereas the oxygen-containing analogue retained greater lung-cancer selectivity. The O→S exchange increased size, polarizability, and lipophilic surface without changing the overall molecular topology, making this pair particularly informative: heteroatom identity influenced tumor-type response rather than producing a uniform increase in cytotoxicity. A similarly restricted interpretation is required for N-methylation in the bis(indolyl) series, which improved activity toward LNCaP cells but did not establish a general advantage across all models. These findings show that methylation and heteroatom replacement can tune membrane passage and local target contacts, but their effects remain dependent on the biochemical context of the selected cancer cells.

O-heterocycles reveal a different structural logic dominated by rigidity, planarity, and conjugation. Fusion of chromene with pyran or pyrimidine rings restricts conformational freedom and creates an extended aromatic surface, while cyano, amino, and imino groups provide additional directional interactions. Halogenated phenyl substituents further reinforce hydrophobic recognition and cellular penetration, explaining their recurring association with enhanced activity in the benzo[f]chromene series. Yet the absence of a validated target prevents this improvement from being assigned specifically to stronger protein binding. In furan–chalcone systems, the furan ring and conjugated enone generate a polarized, nearly planar framework compatible with kinase-binding hypotheses, including EGFR/CDK2 modulation. The same enone may also introduce nonspecific electrophilic reactivity, however, and target selectivity cannot be inferred from docking alone.

The coumarin–thiadiazole compounds provide the strongest evidence that structural affinity and cellular efficacy are not interchangeable. Their planar coumarin surface and heteroatom-rich thiadiazole region favored DNA interaction, but the derivatives displaying the greatest DNA-binding response were not those producing the strongest cytotoxicity. The appended groups therefore controlled more than nucleic-acid recognition; they also influenced uptake, intracellular distribution, or interaction with other targets. Comparable mismatches occur between GLI1 suppression and quinoline cytotoxicity and between tubulin inhibition and indole activity.

Future SAR analysis should therefore be built around controlled structural matrices rather than comparisons between loosely related compounds. Halogen series should be evaluated as matched F/Cl/Br analogues, hydrazones should be compared with the corresponding hydrazides, reduced linkers, and carbonyl analogues, and flexible compounds should be examined alongside conformationally restricted derivatives. For each change, biochemical target inhibition must be measured together with cellular potency, selectivity, permeability, and metabolic stability. Such an approach would transform functional groups into prospective design variables, allowing the next heterocyclic series to be constructed for a defined molecular target and a credible path toward therapeutic development.

## 5 Conclusion

The presented body of evidence clearly indicates that the antitumor activity of N- and O-containing heterocycles does not represent a conceptual breakthrough in itself, as these scaffolds have long been embedded in the foundation of anticancer drug development. Rather than redefining the field, the studies analyzed herein illuminate its current trajectory, where the focus has decisively shifted toward the rational refinement and functional evolution of established heterocyclic frameworks. In this sense, the significance of recent research lies not in the discovery of activity *per se*, but in the increasingly sophisticated strategies employed to enhance selectivity, potency, and mechanistic precision in targeting malignant cells. Across both nitrogen- and oxygen-containing systems, a coherent and compelling pattern emerges the systematic incorporation of hydrazide and hydrazone fragments as key structural determinants of biological performance. Far from being auxiliary modifications, these moieties consistently act as pharmacophoric enhancers, enabling greater conformational adaptability and more effective interaction with critical intracellular targets. The repeated observation of amplified antiproliferative effects, reinforced apoptosis induction, and improved selectivity strongly suggests that hydrazide–hydrazone functionalization has evolved into a central design principle rather than a peripheral modification.

Within nitrogen-containing heterocycles, compound 17 (Acar Çevik et al., 2018) emerges as a particularly striking example of this paradigm, demonstrating submicromolar cytotoxicity with GI_50_ values in the range of 0.01–0.1 μM, thereby illustrating the upper limits of achievable antiproliferative efficiency within this class. In parallel, among oxygen-containing systems, compound 40 (Alblewi et al., 2019) stands out as one of the most potent representatives, exhibiting IC_50_ values of 0.45 μg/mL against MCF-7, 0.7 μg/mL against HCT-116, and 1.7 μg/mL against HepG-2, confirming the remarkable therapeutic promise of structurally refined chromene-based architectures.

Altogether, these findings point toward a compelling and forward-looking conclusion, that N- and O-heterocyclic systems, particularly when integrated with hydrazide–hydrazone fragments and strategically optimized substituents, should be regarded as core scaffolds for the next-generation of anticancer agents. Their consistent and reproducible enhancement of biological activity positions them not merely as subjects of ongoing investigation, but as foundational elements in the design of future therapeutics. The logical continuation of this approach lies in the deliberate prioritization of such frameworks in synthetic strategies, where their structural versatility and pharmacological potential can be further harnessed to address the persistent challenges of efficacy, selectivity, and resistance in modern oncology.

## References

[B1] Abd El-Lateef H. M. Bafail D. Alhalees N. H. Y. (2024). Synthesis, characterization and biological research of novel 2-(quinoline-4-carbonyl)hydrazide-acrylamide hybrids as potential anticancer agents. RSC Adv. 14 (32), 23495–23504. 10.1039/d4ra03963g 39071480 PMC11273260

[B2] Acar Çevik U. Sağlık B. Ardıç C. Özkay Y. Atlı Ö. (2018). Synthesis and evaluation of new benzimidazole derivatives with hydrazone moiety as anticancer agents. Turkish J. Biochem. 43 (2), 151–158. 10.1515/tjb-2017-0167

[B3] Akdağ K. Tok F. Karakuş S. Erdoğan Ö. Çevik Ö. Kaymakçıoğlu B. (2022). Synthesis and biological evaluation of hydrazide-hydrazone derivatives. Acta Chim. Slov. 69 (4), 863–875. 10.17344/acsi.2022.7614 36562164

[B4] Al-Ghorbani M. Thirusangu P. Gurupadaswamy H. D. Vigneshwaran V. Mohammed Y. H. Prabhakar B. T. (2017). Synthesis of novel morpholine conjugated benzophenone analogues and evaluation of antagonistic role against neoplastic development. Bioorg. Chem. 71, 55–66. 10.1016/j.bioorg.2017.01.011 28139247

[B5] Alblewi F. F. Okasha R. M. Hritani Z. M. Mohamed H. M. El-Nassag M. A. A. Halawa A. H. (2019). Antiproliferative effect, cell cycle arrest and apoptosis generation of novel synthesized anticancer heterocyclic derivatives based on 4H-benzo[h]chromene. Bioorg. Chem. 87, 560–571. 10.1016/j.bioorg.2019.03.059 30928878

[B6] Altowyan M. S. Soliman S. M. Haukka M. Al-Shaalan N. H. Alkharboush A. A. Barakat A. (2022). Synthesis, characterization, and cytotoxicity of new spirooxindoles. ACS Omega 7 (40), 35743–35754. 10.1021/acsomega.2c03790 PMC9558703 36249408

[B7] Amin K. M. Taha A. M. George R. F. Mohamed N. M. Elsenduny F. F. (2018). Synthesis, antitumor activity evaluation, and DNA-binding study of coumarin-based agents. Arch. Pharm. 351 (1). 10.1002/ardp.201700199 29148081

[B8] Arafa R. K. Hegazy G. H. Piazza G. A. Abadi A. H. (2013). Synthesis and *in vitro* antiproliferative effect of novel quinoline-based potential anticancer agents. Eur. J. Med. Chem. 63, 826–832. 10.1016/j.ejmech.2013.03.008 23584545

[B9] Banikazemi Z. Mirazimi S. M. Dashti F. Mazandaranian M. R. Akbari M. Morshedi K. (2021). Coumarins and gastrointestinal cancer: a new therapeutic option. Front. Oncol. 11, 752784. 10.3389/fonc.2021.752784 34707995 PMC8542999

[B10] Bashandy M. S. Al-Said M. S. Al-Qasoumi S. I. Ghorab M. M. (2011). Design and synthesis of some novel hydrazide, 1,2-dihydropyridine, chromene derivatives carrying biologically active sulfone moieties with potential anticancer activity. Arzneim. 61 (9), 521–526. 10.1055/s-0031-1296238 22029229

[B11] Berillo D. A. Dyusebaeva M. A. (2022). Synthesis of hydrazides of heterocyclic amines and their antimicrobial and spasmolytic activity. Saudi Pharm. J. 30 (7), 1036–1043. 10.1016/j.jsps.2022.04.009 35903529 PMC9315279

[B12] Bingul M. Tan O. Gardner C. R. Sutton S. K. Arndt G. M. Marshall G. M. (2016). Synthesis, characterization and anti-cancer activity of hydrazide derivatives incorporating a quinoline moiety. Molecules 21 (7), 916. 10.3390/molecules21070916 27428941 PMC6273134

[B13] Bukhari S. N. A. Ejaz H. Elsherif M. A. Junaid K. Zaki I. Masoud R. E. (2022). Design and synthesis of new furan-based derivatives and evaluation of *in vitro* cytotoxic activity. Molecules 27 (8), 2606. 10.3390/molecules27082606 35458804 PMC9024937

[B14] Das Mukherjee D. Kumar N. M. Tantak M. P. Das A. Ganguli A. Datta S. (2016). Development of novel bis(indolyl)-hydrazide-hydrazone derivatives as microtubule-targeting cytotoxic agents. Biochemistry 55 (21), 3020–3035. 10.1021/acs.biochem.5b01127 27110637

[B15] Demurtas M. Baldisserotto A. Lampronti I. (2019). Indole derivatives as multifunctional drugs. Bioorg. Chem. 85, 568–576. 10.1016/j.bioorg.2019.02.007 30825715

[B16] Dyusebaeva M. A. Kalugin S. N. (2015). Thiosemicarbazides of piperidylacetic acid in the synthesis of bisheterocyclic compounds. Russ. J. General Chem. 85, 1775–1778. 10.1134/S107036321507035X

[B17] Dyusebaeva M. A. Elibaeva N. S. Kalugin S. N. (2017). Antimicrobial activity of 1,2,5-trimethylpiperidin-4-ol derivatives. Pharm. Chem. J. 51, 550–552. 10.1007/s11094-017-1652-x

[B18] El-Agrody A. M. Fouda A. M. Al-Dies A. A. M. (2014). Studies on the synthesis and *in vitro* antitumor activity of benzochromene derivatives. Med. Chem. Res. 23, 3187–3199. 10.1007/s00044-013-0904-x

[B19] El-Agrody A. M. Fouda A. M. Assiri M. A. Mora A. Ali T. E. Alam M. M. (2020). *In vitro* anticancer activity of pyrano[3,2-c]chromene derivatives with both cell cycle arrest and apoptosis induction. Med. Chem. Res. 29, 617–629. 10.1007/s00044-019-02494-3

[B20] El-Wahab A. H. F. A. Borik R. M. Al-Dies A. M. Fouda A. M. Mohamed H. M. El-Eisawy R. A. (2024). Targeted potent antimicrobial and antitumor oxygen-heterocyclic-based pyran analogues. Sci. Rep. 14, 9862. 10.1038/s41598-024-59193-2 38684707 PMC11058275

[B21] Fathy U. Azzam M. A. Mahdy F. El-Maghraby S. Allam R. M. (2020). Synthesis and *in vitro* anticancer activity of some novel tetrahydroquinoline derivatives bearing pyrazol and hydrazide moiety. J. Heterocycl. Chem. 57, 2108–2120. 10.1002/jhet.3930

[B22] Galayev O. Garazd Y. Garazd M. Lesyk R. (2015). Synthesis and anticancer activity of 6-heteroarylcoumarins. Eur. J. Med. Chem. 105, 171–181. 10.1016/j.ejmech.2015.10.021 26491980

[B23] Ghorab M. M. Al-Said M. S. (2012). Synthesis and antitumor activity of some novel hydrazide, 1,2-dihydropyridine, chromene, and benzochromene derivatives. J. Heterocycl. Chem. 49, 272–280. 10.1002/jhet.829

[B24] Grande F. Yamada R. Cao X. Aiello F. Garofalo A. Neamati N. (2009). Synthesis and biological evaluation of novel hydrazide based cytotoxic agents. Expert Opin. Investigational Drugs 18 (5), 555–568. 10.1517/13543780902858815 19388873

[B25] Gupta S. Park S. E. Mozaffari S. El-Aarag B. Parang K. Tiwari R. K. (2023). Design, synthesis, and antiproliferative activity of benzopyran-4-one-isoxazole hybrids. Molecules 28 (10), 4220. 10.3390/molecules28104220 37241960 PMC10224329

[B26] Hagbani T. A. Moin A. Hussain T. Gupta N. V. Alshammari F. Rizvi S. M. D. (2023). Anticancer activity of anti-tubercular compound(s) designed on pyrrolyl benzohydrazine scaffolds: a repurposing study. Processes 11 (7), 1889. 10.3390/pr11071889

[B27] Han M. I. Bekçi H. Uba A. I. Yıldırım Y. Karasulu E. Cumaoğlu A. (2019). Synthesis, molecular modeling, *in vivo* study, and anticancer activity of 1,2,4-triazole containing hydrazide-hydrazones derived from (S)-naproxen. Arch. Pharm. 352 (6), e1800365. 10.1002/ardp.201800365 31115928

[B28] Ismail M. M. Rateb H. S. Hussein M. M. (2010). Synthesis and docking studies of novel benzopyran-2-ones with anticancer activity. Eur. J. Med. Chem. 45 (9), 3950–3959. 10.1016/j.ejmech.2010.05.050 20580139

[B29] Kilic-Kurt Z. Acar C. Ergul M. (2020). Novel indole hydrazide derivatives: synthesis and antiproliferative activities. Arch. Pharm. 353 (8), e2000059. 10.1002/ardp.202000059 32419228

[B30] Korcz M. Sączewski F. Bednarski P. J. Kornicka A. (2018). Synthesis, structure, chemical stability, and *in vitro* cytotoxic properties of novel quinoline-3-carbaldehyde hydrazones. Molecules 23 (6), 1497. 10.3390/molecules23061497 29925826 PMC6100353

[B31] Kumar D. Maruthi Kumar N. Ghosh S. Shah K. (2012). Novel bis(indolyl)hydrazide-hydrazones as potent cytotoxic agents. Bioorg. and Med. Chem. Lett. 22 (1), 212–215. 10.1016/j.bmcl.2011.11.031 22123320

[B32] Kumar D. Singh G. Sharma P. Qayum A. Mahajan G. B. (2018). 4-aryl/heteroaryl-4H-fused pyrans as anti-proliferative agents. Anti-Cancer Agents Med. Chem. 18 (1), 57–73. 10.2174/1871520617666170918143911 28925877

[B33] Kumar A. Singh A. K. Singh H. Vijayan V. Kumar D. Naik J. (2023). Nitrogen containing heterocycles as anticancer agents: a medicinal chemistry perspective. Pharmaceuticals 16 (2), 299. 10.3390/ph16020299 37259442 PMC9965678

[B34] Mahmoud M. R. Abu El-Azm F. S. M. Ismail M. F. Hekal M. H. Ali Y. M. (2018). Synthesis and antitumor evaluation of novel tetrahydrobenzo[4′,5′]thieno[3′,2′:5,6]pyrimido[1,2-b]isoquinoline derivatives. Synth. Commun. 48 (4), 428–438. 10.1080/00397911.2017.1406520

[B35] Manetti F. Maresca L. Crivaro E. Pepe S. Cini E. Singh S. (2022). Quinolines and oxazino-quinoline derivatives as small molecule GLI1 inhibitors identified by virtual screening. ACS Med. Chem. Lett. 13 (8), 1329–1336. 10.1021/acsmedchemlett.2c00249 35978701 PMC9377010

[B36] Maresca L. Crivaro E. Migliorini F. Anichini G. Giammona A. Pepe S. (2023). Targeting GLI1 and GLI2 with small molecule inhibitors to suppress GLI-dependent transcription and tumor growth. Pharmacol. Res. 195, 106858. 10.1016/j.phrs.2023.106858 37473878

[B37] Mohamed M. K. Salem E. M. S. Ali I. A. I. (2023). Synthesis and study of anticancer and antioxidant activities of 2-oxoquinoline hydrazide derivatives. Makara J. Sci. 27 (1). 10.7454/mss.v27i1.1495

[B38] Nagender P. Naresh Kumar R. Malla Reddy G. Krishna Swaroop D. Poornachandra Y. Ganesh Kumar C. (2016). Synthesis of novel hydrazone and azole functionalized pyrazolo[3,4-b]pyridine derivatives as promising anticancer agents. Bioorg. and Med. Chem. Lett. 26 (18), 4427–4432. 10.1016/j.bmcl.2016.08.006 27528432

[B39] Nasr T. Bondock S. Youns M. (2014). Anticancer activity of new coumarin substituted hydrazide-hydrazone derivatives. Eur. J. Med. Chem. 76, 539–548. 10.1016/j.ejmech.2014.02.026 24607878

[B40] Onnis V. Cocco M. T. Fadda R. Congiu C. (2009). Synthesis and evaluation of anticancer activity of 2-arylamino-6-trifluoromethyl-3-(hydrazonocarbonyl)pyridines. Bioorg. and Med. Chem. 17 (17), 6158–6165. 10.1016/j.bmc.2009.07.066 19679483

[B41] Rani S. Kumar D. Kamra N. Thakral S. Singh A. Sangwan P. L. (2022). Design and synthesis of highly oxygenated furo[3,2-c]pyran4-ones and furo[3,2-c]chromen-4-ones scaffold as anticancer and antimicrobial agents. J. Heterocycl. Chem. 59 (1), 144–160. 10.1002/jhet.4374

[B42] Refat H. M. Fadda A. A. (2015). Synthesis and cytotoxicity activity of some novel hydrazide, pyrazole, isoxazole, pyrimidine and fused pyran-2-one derivatives. Heterocycles 91, 1212–1226. 10.3987/com-15-13209

[B43] Salama E. E. Youssef M. F. Aboelmagd A. (2023). Discovery of potent indolyl-hydrazones as kinase inhibitors for breast cancer. Pharmaceuticals 16 (12), 1724. 10.3390/ph16121724 PMC10748079 38139850

[B44] Samir M. Mohareb R. M. (2022). Novel synthesis of pyran-3-hydrazide derivatives and their uses to the synthesis hydrazide-hydrazone, pyrazole and thiazole derivatives with anticancer activities. Bull. Chem. Soc. Ethiop. 35 (3), 573–586. 10.4314/bcse.v35i3.9

[B45] Sarhan A. A. M. Boraei A. T. A. Barakat A. Nafie M. S. (2020). Discovery of hydrazide-based pyridazino[4,5-b]indole scaffold as a new phosphoinositide 3-kinase (PI3K) inhibitor for breast cancer therapy. RSC Adv. 10 (33), 19534–19541. 10.1039/d0ra02798g 35515454 PMC9054070

[B46] Saruengkhanphasit R. Butkinaree C. Ornnork N. (2021). Identification of new indole carbohydrazide derivatives as tubulin inhibitors. Bioorg. Chem. 110, 104795. 10.1016/j.bioorg.2021.104795 33730670

[B47] Şenkardeş S. Türe A. Ekrek S. Durak A. T. Abbak M. Çevik Ö. (2021). Novel 2,6-disubstituted pyridine hydrazones: synthesis, anticancer activity, docking studies and effects on caspase-3-mediated apoptosis. J. Mol. Struct. 1223, 128962. 10.1016/j.molstruc.2020.128962

[B48] Shahbaz M. Perween A. Momal U. Imran M. Ul Hassan M. H. Naeem H. (2024). Recent perspectives on anticancer potential of coumarin against different human malignancies: an updated review. Food Sci. and Nutr. 13 (1), e4696. 10.1002/fsn3.4696 39803273 PMC11717051

[B49] Solomon V. R. Lee H. (2012). Anti-breast cancer activity of heteroaryl chalcone derivatives. Biomed. and Pharmacother. 66 (3), 213–220. 10.1016/j.biopha.2011.11.013 22440895

[B50] Sreenivasulu R. Sujitha P. Jadav S. S. Ahsan M. J. Kumar C. G. Raju R. R. (2017). Synthesis and antitumor evaluation of indole–indazolyl hydrazide–hydrazone derivatives. Monatsh. für Chem. 148, 305–314. 10.1007/s00706-016-1750-6

[B51] Sundaree S. Vaddula B. R. Tantak M. P. Khandagale S. B. Shi C. Shah K. (2016). Synthesis and anticancer activity study of indolyl hydrazide–hydrazones. Med. Chem. Res. 25, 941–950. 10.1007/s00044-016-1522-1

[B52] Taha M. Shah S. A. A. Afifi M. Zulkeflee M. Sultan S. Wadood A. (2017). Morpholine hydrazone scaffold: synthesis, anticancer activity and docking studies. Chin. Chem. Lett. 28 (3), 607–611. 10.1016/j.cclet.2016.10.020

[B53] Vladimirova S. Hristova R. Iliev I. (2024). Synthesis, cytotoxicity and antiproliferative effect of new pyrrole hydrazones. Molecules 29 (23), 5499. 10.3390/molecules29235499 39683659 PMC11643554

[B54] Yancheva D. Argirova M. Georgieva I. Milanova V. Guncheva M. Rangelov M. (2024). Antiproliferative and pro-apoptotic activity and tubulin dynamics modulation of 1H-benzimidazol-2-yl hydrazones in human breast cancer cell line MDA-MB-231. Molecules 29 (10), 2400. 10.3390/molecules29102400 38792260 PMC11123699

[B55] Yu V. Ten A. Baktybayeva L. Sagatbekova I. Praliyev K. Zolotareva D. (2018). Synthesis and biological evaluation of 1,3,8-triazaspiro[4.5]decane-2,4-dione derivatives as myelostimulators. J. Chem. 2018, 7346835–7346839. 10.1155/2018/7346835

[B56] Yu V. K. Sycheva Y. S. Kairanbayeva G. K. Dembitsky V. M. Balabekova M. K. Tokusheva A. N. (2023). Naphthaleneoxypropargyl-containing piperazine as a regulator of effector immune cell populations upon an aseptic inflammation. Molecules 28 (20), 7023. 10.3390/molecules28207023 37894502 PMC10608911

[B57] Yu V. Balabekova M. Ten A. Zharkynbek T. Koks S. Alimova M. (2025). Targeted restoration of T-cell subsets by a fluorinated piperazine derivative β-cyclodextrin complex in experimental pulmonary inflammation. Molecules 30 (13), 2741. 10.3390/molecules30132741 40649259 PMC12250881

[B58] Zebbiche Z. Tekin S. Küçükbay H. Yüksel F. Boumoud B. (2021). Synthesis and anticancer properties of novel hydrazone derivatives incorporating pyridine and isatin moieties. Arch. Pharm. 354 (5), e2000377. 10.1002/ardp.202000377 33368627

